# Green Corrosion Inhibition on Carbon-Fibre-Reinforced Aluminium Laminate in NaCl Using Aerva Lanata Flower Extract

**DOI:** 10.3390/polym14091700

**Published:** 2022-04-21

**Authors:** Navasingh Rajesh Jesudoss Hynes, Nagarajan Jawahar Vignesh, Claudia Barile, Pitchumani Shenbaga Velu, Thangagiri Baskaran, Jebas Thangiah Winowlin Jappes, Omar Ali Al-Khashman, Michail Brykov, Antoaneta Ene

**Affiliations:** 1 Department of Mechanical Engineering, Mepco Schlenk Engineering College, Sivakasi 626005, Virudhunagar, India; vigneshmech@mepcoeng.ac.in; 2 Dipartimento di Meccanica, Matematica e Management, Politecnico di Bari, Viale Japigia 182, 70126 Bari, Italy; claudia.barile@poliba.it; 3 Department of Mechanical Engineering, PSR Engineering College, Sivakasi 626140, Virudhunagar, India; velupitchumani@gmail.com; 4 Department of Chemistry, Mepco Schlenk Engineering College, Sivakasi 626005, Virudhunagar, India; thangagiri@gmail.com; 5 School of Automotive and Mechanical Engineering, Kalasalingam Academy of Research and Education, Krishnankoil 626126, Virudhunagar, India; winowlin@yahoo.com; 6 Department of Environmental Engineering, Faculty of Engineering, Al-Hussein Bin Talal University, Ma’an P.O. Box 20, Jordan; omarkhashman@yahoo.com; 7 Zaporizhzhya Polytechnic National University, 69600 Zaporizhzhya, Zaporiz′ka Oblast′, Ukraine; m@brykov.com; 8 Department of Chemistry, Physics, and Environment, INPOLDE Research Center, Dunarea de Jos University of Galati, 800008 Galaţi, Romania

**Keywords:** *Aerva lanata*, green corrosion inhibitor, carbon-fibre-reinforced aluminium laminates, electrochemical-impedance spectroscopy, polarization measurements, SEM study, langmuir absorption technique

## Abstract

Aluminium-based fibre–metal laminates are lucrative candidates for aerospace manufacturers since they are lightweight and high-strength materials. The flower extract of aerva lanata was studied in order to prevent the effect of corrosion on the aluminium-based fibre–metal laminates (FMLs) in basic media. It is considered an eco-friendly corrosion inhibitor using natural sources. Its flower species belong to the Amaranthaceae family. The results of the Fourier-transform infrared spectroscopy (FTIR) show that this flower extract includes organic compounds such as aromatic links, heteroatoms, and oxygen, which can be used as an organic corrosion inhibitor in an acidic environment. The effectiveness of the aerva-lanata flower behaviour in acting as an inhibitor of the corrosion process of FMLs was studied in 3.5% NaCl solution. The inhibition efficiency was calculated within a range of concentration of the inhibitor at room temperature, using the weight-loss method, potentiodynamic polarization measurements and electrochemical-impedance spectroscopy (EIS). The results indicate a characterization of about 87.02% in the presence of 600 ppm of inhibitor. The Tafel curve in the polarization experiments shows an inhibition efficiency of 88%. The inhibition mechanism was the absorption on the FML surface, and its absorption was observed with the aid of the Langmuir adsorption isotherm. This complex protective film occupies a larger surface area on the surface of the FML. Hence, by restricting the surface of the metallic layer from the corrosive medium, the charge and ion switch at the FML surface is reduced, thereby increasing the corrosion resistance.

## 1. Introduction

Nowadays, the great part of the automotive and aerospace industries is searching for improvements to the use of lightweight components in order to increase the strength-to-density ratio, which implies the possibility of decreasing the weight of structures while simultaneously ensuring the high performance in terms of strength, stiffness, flexibility, corrosion resistance, wear resistance, etc. The most famous automobile companies are looking for hybrid materials that could assist in achieving the above-mentioned properties [1,2]. Friction stud welding [3], diffusion bonding [4], friction welding [5], friction drilling, and friction riveting are some of the processes employed for joining multi-material structures. Nevertheless, multi-material structures developed by combining fibres and metal laminates have promising properties and applications. An analysis of new works shows that sufficiently advanced protection methods, such as arc spraying with flux-cored wire [6], novel superhydrophobic coatings [7], and superdispersed polytetrafluoroethylene (SPTFE) and polyvinylidene fluoride (PVDF) coatings [8] can be used for various connected structural elements, including aluminium alloys, which makes them promising methods for the aerospace industry.

Carbon-fibre-reinforced plastics (CFRPs) are characterised by the best specific strength that is at least two times higher than steel. The weight of a component could be decreased by up to half if a CFRP is used instead of steel. For vehicle applications, this means the possibility of making lighter vehicles that consume less fuel. As a result, they are considered advanced structural materials [9].

Currently, the design of Al-based fibre–metal laminates is restricted to the use of glass fibre (GF), since the risk of galvanic corrosion prohibits the use of carbon-fibre-reinforced plastics.

Previous research has explored galvanic corrosion between fibre composites and a variety of metals. Tavakkolizadeh et al. [9] suggested that galvanic corrosion occurs when carbon fibres have been in electrical contact with steel in an electrolyte. Moreover, they have shown that the use of coating could reduce the rate of galvanic corrosion, but not eliminate it. Ireland et al. studied the effect of carbon nanotubes, including GF-reinforced epoxy, on galvanic corrosion with aluminium. They assessed that galvanic corrosion did occur between them, even if a polymer barrier existed between the two conductive materials.

Plant extracts are highly available natural products that have corrosion-inhibition properties and are a renewable source and by nature, they are non-poisonous. The ample chemical constituents that are present in the plant extracts such as alkenes, polyphenols, and aromatics are capable of inhibiting the corrosion process in mild steel [10]. Most of the herbal products containing functional groups such as C–Cl, C–O, NH_2_, C–H, C=O, O–H and CHO are potential inhibitors. The above compounds become adsorbed and form a protective layer on the surface of the steel to restrict the formation of corrosion.

Steven et al. [11] investigated the probability of galvanic corrosion of carbon fibre and aluminium in FML. In this examination, the authors studied the galvanic corrosion behaviour between the bulk metallic glass (BMG) and the CFRP. They assessed that the BMG showed less corrosion than the Al combined with CFRP.

Mehdi Yari et al. [12] investigated the properties of carbon composites. The authors determined that when a considerable region of carbon composites is coupled to small metallic parts such as nuts, screws and clasps, the galvanic corrosion was a significant threat.

Perez et al. [13] researched the galvanic corrosion between carbon steel and a hardened metal that was treated with 1M NaOH. The evaluation was carried out at two different conditions: with and without chloride. They determined that there is no significant threat or massive damage due to galvanic corrosion when carbon steel and hardened metal are electrically coupled in a strong, fortified structure.

Akhil et al. [14] investigated the corrosion behaviour of mild steel in 0.5 M H_2_SO_4_ by using Saraka Ashoka. They proposed that the application of this extract containing epicatechin helps in limiting the corrosion rate of the mild steel. The excellent inhibition effect of mild metal in 0.5 M H_2_SO_4_ was evaluated at 100 mg/L by using the electrochemical and weight-loss measurements. Atomic force microscopy (AFM) and scanning electron microscopy (SEM) were also used for analysing the surface morphology. The electrochemical studies showed that there was a 95.48% inhibition effectivity at 100 mg/L inhibitor concentration. The following Table 1 describes the inhibition efficiencies exhibited by different plant extracts and the corresponding medium used during the process [15,16,17,18,19,20,21,22,23,24,25,26,27,28,29,30].

Jakubczak et al. [15] carried out their study on the interlaminar shear strength of the CARALLs with different aluminium-surface preparations and various fibre combinations because of the effect of thermal ageing. Their study revealed that galvanic corrosion is reduced when there is an insertion of a thin glass ply in between the metal sheets and carbon laminates. However, this insertion does not have any effect on the ILSS or the thermal fatigue of the laminates. Pan et al. performed a study on the influence of anodizing and annealing the aluminium sheets in an FML [16]. They found that the mechanical properties lowered due to the effect of annealing. Kim et al. (2010) carried out a systematic investigation on the adhesion strength of the CFRP/steel bond to determine the effect of the surface morphology of steel. They incorporated a micro-periodic line pattern on the steel surface for carrying out the investigation. Their findings show that the enhancement of strength is due to the transition from interfacial failure to cohesive failure. Reyes and Gupta (2009) included glass fibre-reinforced polypropylene in place of conventional thermosetting parts used in FMLs. They applied a zinc coating to the surface of the steel layer to achieve very good adhesion on the polymer–metal interface. Carbon-fibre-reinforced aluminium laminates were used for carrying out the low-velocity impact on the specimens. Experimental and numerical simulations were performed on the prepared samples, both qualitatively and quantitatively. It was found that matrix fractures, carbon-fibre cracking, and delamination were the important modes of damage [31]. Novel superhydrophobic coatings were applied to the surface of an aluminium alloy to prevent the effect of corrosion by using Al_2_O_3_/siloxane hybrids that were grown in situ on the surface of the aluminium. The experiment proved that the aluminium alloy AA 2024 has excellent corrosion resistance in NaCl, alkaline and other acidic environments [32]. Superdispersed polytetrafluoroethylene (SPTFE) and polyvinylidene fluoride (PVDF) were used as the coating medium for studying the anti-icing properties of the samples, which were oxidised by plasma electrolytic oxidation. The coatings having the combined PVDF–SPFTE layers with a ratio of 1:4 showed significant performance with hydrophobicity, ice-phobic and electrochemical characteristics compared to all the other sample combinations. The use of this PVDF–SPFTE coating also proved that there was a reduction in the corrosion current density in powers of order 5 when compared with uncoated aluminium alloy [32]. The cored-wire arc-spraying technique was used for spraying ultra-high-molecular-weight polyethene (UHMWPE) particles onto the surface of aluminium during the aluminium spray coatings. These particles act as sealants. The microstructure evaluation was carried out in the study. To study the effect of corrosion, neutral-salt spraying and electrochemical analysis were performed on the coated aluminium sample. The study revealed that the UHMWPE particles acted as sealants, helping to improve corrosion resistance [33].

The present paper aims to study the behaviour of aerva lanata in aluminium-based FMLs, in terms of corrosion and absorption. It is an Indian plant species and belongs to the family of Amaranthaceae. The extraction process was carried out on a soxhlet-extraction handle apparatus with a 3.5% NaCl solution, and its inhibition efficiencies on carbon-fibre-and-aluminium-metal-based FMLs were investigated. The performance and mechanism of inhibition was also discussed. The characteristics of FMLs were studied by employing Fourier-transform infrared spectroscopy (FTIR) and SEM analysis was carried out on the prepared surface.

## 2. Experimentation

### 2.1. Materials Preparation

In the present study, carbon-fibre-and-aluminium-metal-based FMLs were studied. The fibre–metal laminates were made by stacking alternating layers of AA 6061 sheets and carbon fibre for a total of 5 layers (starting from AA 6061 and again ending at AA 6061 with carbon fibre in alternate layers). The dimensions of the sheets and fibres were cut to 150 mm × 250 mm, which was equal to the size of the die. The thickness or depth of the die used was about 5 mm. The thickness of the AA 6061 sheets was equal to 0.5 mm and that of the carbon fibre was 0.25 mm. The elemental composition of AA 6061 is given in the following Table 2.

**Figure 1:**
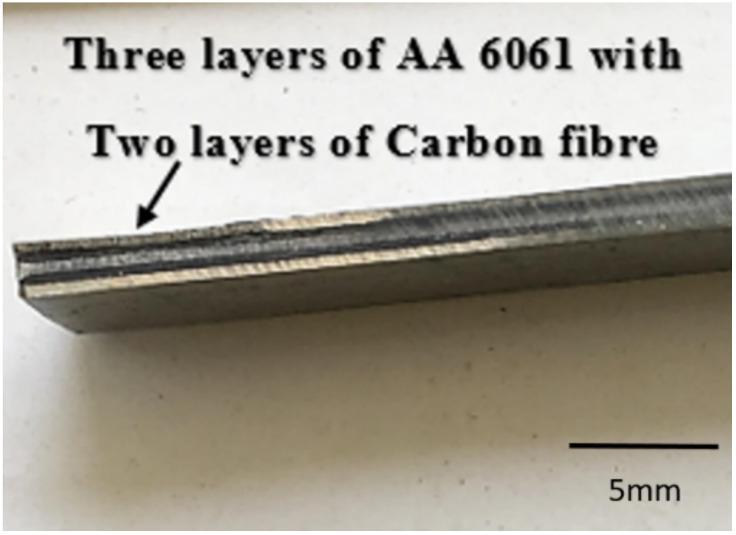
Cross-sectional view of the laminate sandwich.

For preparing these FMLs, carbon fibre and aluminium sheets were bonded together by deposing a layer of reinforcing adhesive. They were joined together by using epoxy resin with the commercial name Araldite LY 556 bought from a retail manufacturer Excel Trading Corporation, Pune, India. The hardener used was Aradur HY 951 bought from a retail manufacturer Aerium Tech Private Limited, Mumbai, India. Both the epoxy resin and the hardener were mixed in the ratio of 10:1 parts by weight using the rule of mixture calculations. Then, a compressive force of about 10 bars was applied to the die using a compressive moulding machine available at Department of Mechanical Engineering, Mepco Schlenk Engineering College, Sivakasi, India. The following Figure 1 shows the prepared FML. After this, for carrying out the corrosion tests, the samples were cut from the FML in dimensions of 5 mm × 5 mm. The following Figure 1 shows the cross-sectional view of the carbon fibre/aluminium 6061 FML laminate sandwich.

The preparation of the flower extract as a corrosion inhibitor is discussed herein. Initially, the *Aerva-lanata* flower was dried for 7 sunny days. Then the extraction process was carried out in a soxhlet-extraction mantle apparatus. A total of 10 g of aerva-lanata-flower powder was mixed with 170 mL of distilled water. The powdered sample was refluxed for four hours using distilled water at 80 °C. Then it was filtered to obtain the extract solution of 70 mL. Finally, the filtered solution was heated on a hot plate. The hot plate was maintained at 100 °C for more than 1 h. A quantity equal to 70 mL was boiled by a hot plate until 40–35 mL remained, then it was poured in petri dish. The petri dish was kept in an open atmosphere for three days. Finally, the *Aerva-lanata*-extract powder was collected.

### 2.2. Weight-Loss Measurements

For evaluating the rate of corrosion in aqueous solutions, the method of immersion represents an easy technique. In the present work, the immersion corrosion tests on FMLs were carried out for a period of 5 days in a medium of 3.5% NaCl. For weight-loss estimation, the carbon-fibre-and-aluminium-metal-based FMLs were prepared according to ASTM G 31-72 standards. Before the FMLs were exposed to the environment, they were cleaned, dried and weighed, and then exposed to 3.5% NaCl. The results were examined at 25 °C in the presence and absence of inhibitor for an immersion period of 5 days. By using this method, the corrosion rate and the efficiency of the inhibitor was also determined.

### 2.3. Electrochemical Measurements

Electrochemical measurements were carried out using the simple three-electrode cell system. It was a Cyclic Voltammetry Electrochemical Cell manufactured by Ossila Ltd., Sheffield, UK. It involved an easier method containing a mild steel electrode, a platinum electrode, and a saturated calomel electrode (SCE) as the working, counter and reference electrodes, respectively. During the tests, the working electrode was immersed in the 3.5% NaCl test solution for 1 h, to obtain a stabilised open-circuit potential (OCP). Electrochemical-impedance spectroscopy (EIS) was used for scanning from 100 kHz to 0.01 Hz frequency, with a sign-amplitude perturbation of 5 mV at OCP. From this value, the Tafel and Nquist graphs were drawn in order to determine the inhibition efficiency of the extracted *Aerva-lanata* sample on the FMLs.

### 2.4. FTIR Spectroscopy

In order to understand the inhibition mechanism in a better way, the FTIR spectra of *Aerva-lanata* extract were examined. The FTIR measurements were carried out on a IRSpirit FTIR Spectrometer, Japan purchased from Toshvin Analytical Pvt. Ltd., Mumbai, India. The *Aerva-lanata* extract was reduced into powder form for FTIR characterization by means of a FTIR 8400s spectrophotometer with a wave number between 500–4000 cm^−1^.

### 2.5. Hardness Studies

The hardness test was used to study the influence of the corrosive NaCl on the hardness of the FMLs. The hardness tests were carried out on a micro Vickers hardness tester purchased from Walter Uhl technische Mikroskopie Gmbh & Co. KG, Asslar, Germany. Tests were carried out on a micro Vickers hardness tester with an indentation load of 500 g for 10 s on the FMLs under three conditions. First, the FML was tested for hardness without being subjected to corrosive NaCl. Secondly, the FML specimen that had not been treated with the *Aerva lanata* extract surface coating was subjected to NaCl corrosion and then measured with Vickers hardness. Finally, the hardness value was also measured for the specimen that was first coated with the *Aerva lanata* extract and then subjected to corrosion.

### 2.6. SEM Imaging

Scanning-electron-microscopic studies were carried out on the prepared samples. The SEM analysis was performed on a ZEISS GeminiSEM Field Emission Scanning Electron Microscope, made in Oberkochen, Germany. Images of the carbon-fibre-reinforced aluminium laminate, the bare material, and the flower-extract-coated specimen, both before and after the immersion test, were obtained.

## 3. Results and Discussion

### 3.1. FTIR Characterization

**Figure 2:**
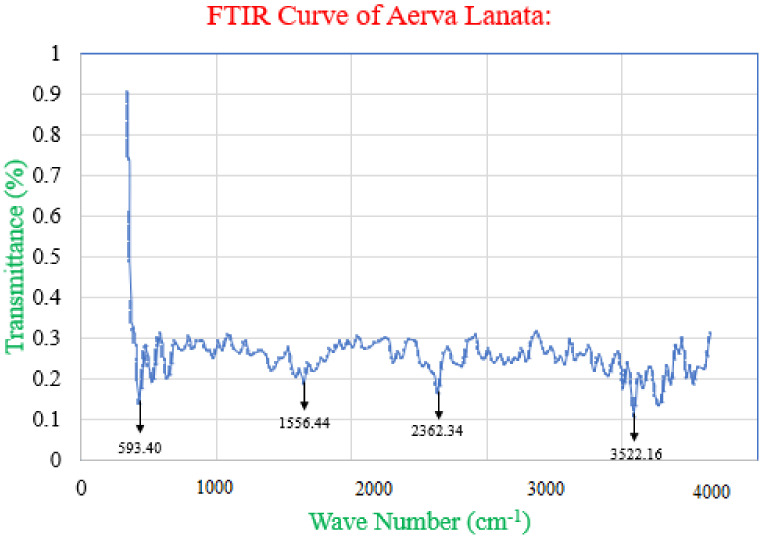
FTIR results of *Aerva lanata*.

Figure 2 reports the FTIR results. It shows that the *Aerva lanata* sample had the stretching vibration of O–H, causing the peak centre at 3522.16 cm^−1^ belonging to the amine functional group [28]. The stretching vibration of C–Cl caused the peak at 2362.34 cm^−1^, which belongs to carbolic-acid functional group. The stretching vibration of C–O caused the peak at 1556.44 cm^−1^, which belongs to useful alkene group. The stretching vibration of C–H caused the peak at 593.40 cm^−1^, which belongs to aromatic functional group. The FTIR results confirm the presence of anticorrosive properties of the *Aerva lanata* extract that prevent corrosion on the surface. Since the extracts are mainly composed of few low-molecular-weight compounds, FTIR analysis is a suitable method to identify them. Therefore, to examine the more prevalent compounds in the *Aerva lanata* extract, FTIR analysis was used.

### 3.2. Weight-Loss Measurements

Table 3 shows the weight-loss values, inhibition efficiency (η %) and surface coverage (θ) for the carbon-fibre FML at different concentrations of *Aerva-lanata* extract. From the weight-loss values, corrosion rates (CR) were calculated by the following equation,

CR = (ΔW × K)/ρ × A × t(1)

where CR is the corrosion rate in mmpy, ΔW is the weight loss before/after the immersion test, K is a constant (for mmpy K value be 8.75 × 10^4^), ρ is the specimen density (g/cm^3^), A is the exposed area (cm^2^) and t is the exposure time (h). The inhibition efficiency (η) was calculated as follows:

(2) $$\eta =\frac{W_{o}-W_{i}}{W_{o}}\times 100$$

where W_i_ and W_0_ are the weight of the specimen in the presence and absence of the inhibitor, respectively.

Table 3 also shows the corrosion rate (mmpy) and the inhibition efficiency (η %) of the FML in 3.5% NaCl at different concentrations of *Aerva-lanata* extract. From the above results, it can be seen that the corrosion rate of the FML was reduced as the concentration of *Aerva-lanata* extract was increased. This is due to the phenomenon of the precipitation reaction caused by the adsorption of the active ingredients of the *Aerva-lanata* extract on the carbon-fibre-and-aluminium-metal surface. The average inhibition effectivity of the *Aerva-lanata* inhibitor on the FML was 87.032%. Table 4 indicates the weight of the specimens before and after coating.

**Figure 3:**
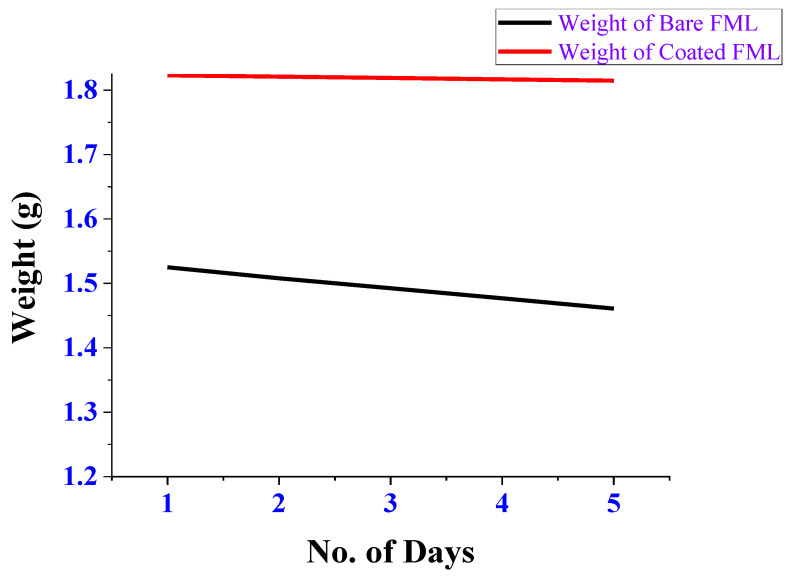
Weight Loss (g) Vs. No. of Days in NaCl.

**Figure 4:**
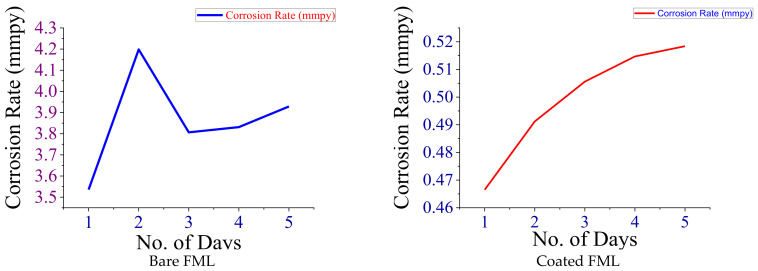
Corrosion rate (mmpy) vs. no. of days in NaCl.

Figure 3 indicates the weight loss of FML due to the action of corrosion with respect to the number of days for each of the bare and coated FML in 3.5% NaCl. Figure 3 indicates that there was only a small reduction in the weight of the coated FML when compared to the uncoated bare FML. So, the *Aerva lanata* extract yields a great efficiency by providing a protective covering over the surface of the FML. Figure 4 indicates the corrosion rate of FML with respect to the number of days in which the FML was immersed in 3.5% NaCl. This *Aerva lanata* extract was capable of yielding an effectivity of about 87% on carbon-fibre-and-aluminium-metal-based FMLs in 3.5% NaCl.

### 3.3. Electrochemical Study

#### 3.3.1. Polarization Measurements

**Figure 5:**
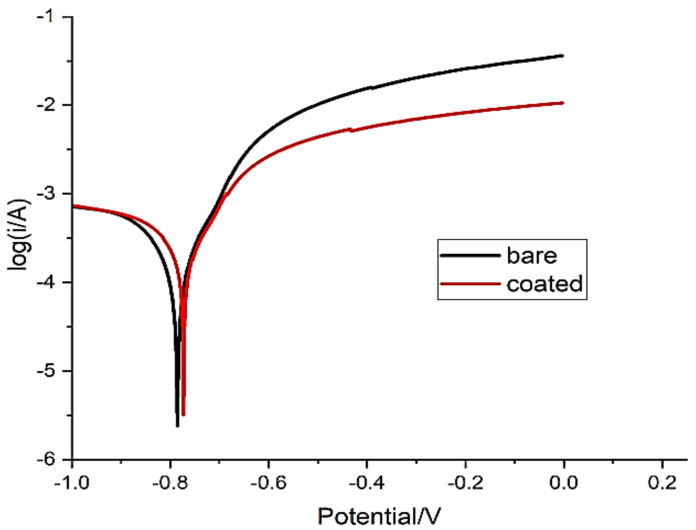
Tafel graph for FML in NaCl.

The effect of the concentration of the *Aerva-lanata* extract on the polarization behaviour of the FML in 3.5% NaCl were analysed and the Tafel plots were recorded for different inhibitor concentrations, as shown in Figure 5. The corrosion current densities were calculated by the intersection corresponding to the corrosion potential. When there was higher concentration, it resulted in a lower current density at 600 ppm [29].

(3) $$\eta =\frac{I_{o\;\mathrm{corr}}-I_{i\;\mathrm{corr}}}{I_{o\;\mathrm{corr}}}$$

where I_o corr_ and I_i corr_ represent the corrosion-density values with and without the inhibitor on the FML surface, respectively.

Figure 5 clearly shows that the anodic metallic-dissolution and cathodic hydrogen-evolution reactions were inhibited when the concentration of aerva lanata was increased in the aggressive medium. The Tafel plots were plotted for the FML for two conditions viz. before and after adding the aerva-lanata-flower extract to the FML surface, which were controlled by means of charge transfer between the cathodic and anodic reaction mechanism. The combination of physisorption and chemisorption properties causes the active ingredients of the *Aerva-lanata*-flower extract to be absorbed strongly onto the FML surface. Corrosion on the surface was prevented by using this flower extract on the surface of the FML, and it can be proved by fact that the corrosion current-density value decreasing by increasing the *Aerva-lanata* inhibitor concentration. The result from Table 4 suggests that by increasing the concentration of the *Aerva-lanata* extract, there is decrease in the corrosion current density. The lowest corrosion current density of 2.335 × 10^−3^ A/cm^2^ was obtained at 600 ppm concentration at the rate of 88% on the carbon-fibre-and-aluminium-metal-based FML. From Figure 5 it can be seen that there was a positive shift, which is due to the corrosion resistance of the carbon-fibre-and-aluminium-metal-based FMLs with the *Aerva-lanata* extract concentration on the surface. It can be seen that the maximum positive shift happens at 600 ppm compared to the other inhibitor concentrations. Additionally, this 600 ppm concentrated solution yielded the highest efficiency of about 88%, which is shown in Table 5.

#### 3.3.2. Electrochemical-Impedance Spectroscopy

**Figure 6:**
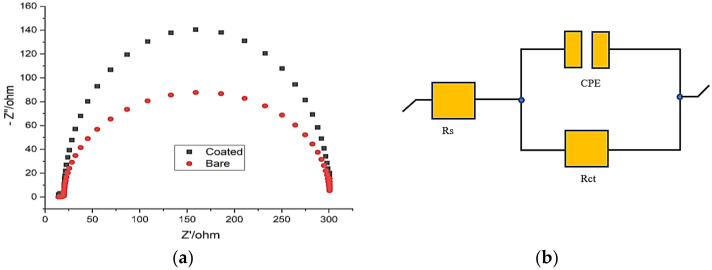
(**a**) Nyquist plot for FML in NaCl; (**b**) Equivalent-circuit for calculation.

Electrochemical-impedance-spectroscopy (EIS) measurements were performed on the carbon-fibre-and-aluminium-metal-based fibre–metal laminates to study the impedance parameters in a 3.5% NaCl environment with different *Aerva-lanata*-flower-extract concentrations. Table 6 shows the results of the EIS process. Figure 6a shows the EIS Nyquist curves with different concentrations of *Aerva-lanata* extract.

It can be seen that as the concentration of *Aerva lanata* extract increased, the values of charge-transfer resistance (Rct) also increased. A maximum value of 301.15 Ω cm^2^ was reached. This rise in charge-transfer resistance reduced the number of active sites created by the adsorption of chloride ions on the surface of the FML, which led to the protection. The equivalent circuit is shown in Figure 6b.

In the Nyquist plot, it can be seen that a semicircle is formed in each curve, which is due to the load-transfer resistance, which stands for a particular time constant. Increasing the concentration of the *Aerva lanata* extract enlarged the capacitive-loop diameter from uncoated to coated at about 600 ppm, from which it is understood that an inhibition effect was being developed. The inhibition efficiency can be calculated in the impedance study using the following formula,

(4) $$\eta =\frac{R_{\mathrm{ct}}-R{{}^{\circ}}_{\mathrm{ct}}}{R_{\mathrm{ct}}}\times 100$$

where R_ct_ and R°_ct_ are the inhibitor and non-inhibitor load-transfer resistances, respectively. The efficiency of inhibition is enhanced by increasing the *Aerva lanata* extract concentration, reaching the highest value of 85.9 percent at 600 mg/L.

Table 6 clearly shows that when the Rct value increases, the CPE value correspondingly increases. This is due to the formation of the protective film on the surface of the carbon-fibre-and-aluminium-metal laminates. This transition in Rct and CPE values was caused by the substitution of water molecules on the carbon-fibre-and-aluminium-metal-based FML surface by the adsorption of the *Aerva lanata* inhibitor. The inhibited solutions had higher values of n than those that were uninhibited due to a reduction in the surface heterogeneity as a result of the adsorption of the electrode electrolyte *Aerva lanata* inhibitor on the FML interface based on carbon fibre and aluminium metal. Inductive loops were present at low frequency for the EIS curves of a blank solution, which is due to the result of the surface relaxation of the adsorbed intermediate products. The inductive loops disappeared for the remaining concentrations.

### 3.4. Mechanical-Hardness Test

**Figure 7:**
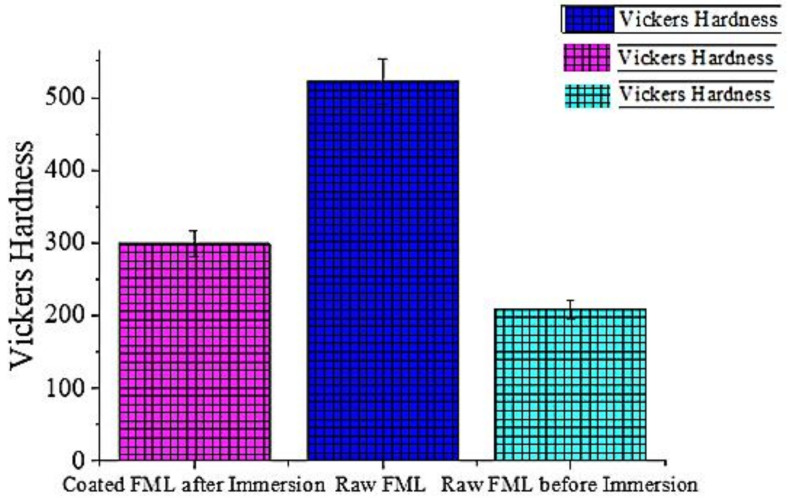
Vickers hardness test on FML coated with *Aerva-lanata* extract.

The Vickers hardness test conducted on the carbon-fibre-and-aluminium-metal laminates that were immersed in the 3.5% NaCl corrosion test solution are shown in Figure 7. The raw specimen indicated a most astounding hardness strength of 522 VHN, whereas when it was subjected to corrosion its hardness value drastically decreased to 209 VHN. However, at the same time, when the corroded FML was treated with the *Aerva lanata* extract, it improved the hardness ability up to 299 VHN. Table 7 shows the Vickers hardness values for the FML at various conditions. 

### 3.5. Surface Analysis

#### Scanning Electron Microscope

**Figure 8:**
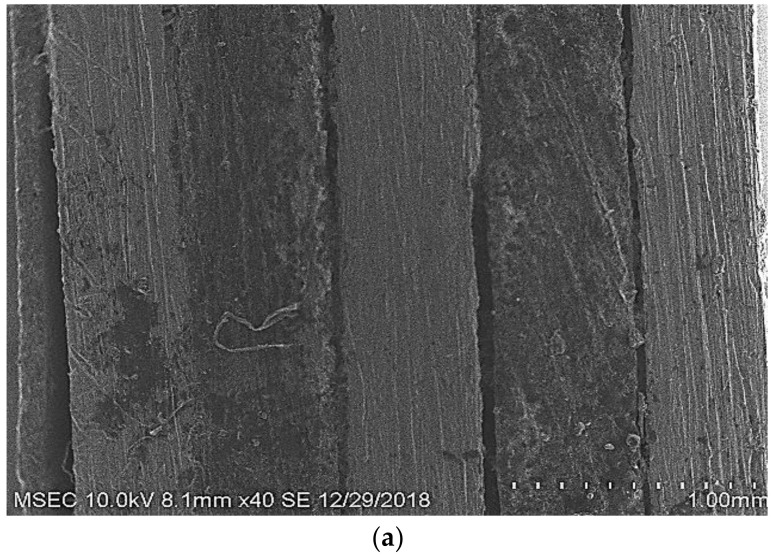

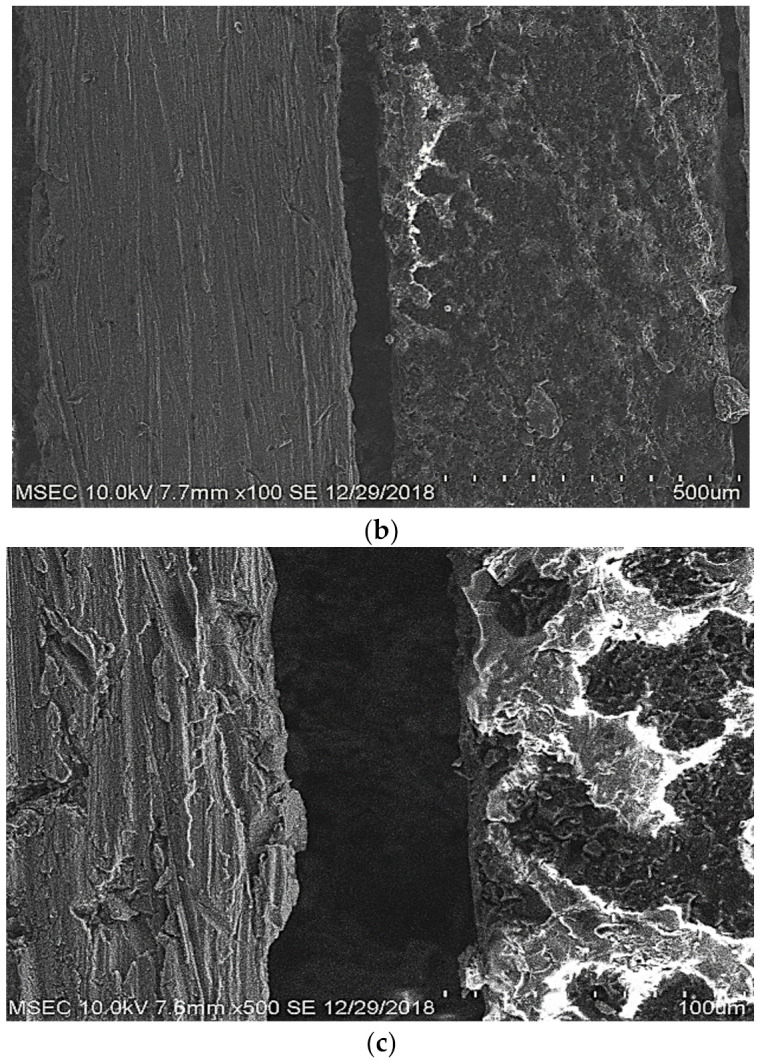
SEM images of carbon-fibre-reinforced aluminium laminate. (**a**) carbon-fibre-reinforced aluminium laminate; (**b**) carbon-fibre-reinforced aluminium laminate after immersion test; (**c**) View of corroded region in carbon-fibre-reinforced aluminium laminate after immersion test.

Figure 8 shows the carbon-fibre-and-aluminium-metal-based fibre–metal laminate surface morphology in 3.5% NaCl solution with and without the aerva-lanata-flower inhibitor. Figure 8a shows the bare carbon-fibre-and-aluminium-metal-based fibre–metal laminates that were subjected to polishing before undergoing SEM, whereas Figure 8b shows the SEM image of the bare carbon-fibre-and-aluminium-metal-based fibre–metal laminate after eight hours of immersion in 3.5% NaCl, and Figure 8c shows the SEM image of the carbon-fibre-and-aluminium-metal-based fibre–metal laminates that were immersed in 3.5% NaCl and also coated with the *Aerva-lanata* extract. The surface morphology of the carbon-fibre-and-aluminium-metal-based fibre–metal-laminate specimen was incredibly rough due to surface corrosion when the specimen was immersed in the 3.5% NaCl solution, but the specimen that was coated with the *Aerva-lanata* extract showed less surface morphology than Figure 8b. This is due to the formation of an extract-solution protective layer on the carbon-fibre-and-aluminium-metal-based fibre–metal-laminate surface. The smoother surface was obtained by the presence of the green corrosion inhibitor on the surface of the carbon-fibre-and-aluminium-metal-based fibre–metal laminates [29]. The effect of microstructure was investigated by comparing the corrosion behaviour of the devitrified (crystalline) to that of the non-crystalline BMG. The results were similar to those reported in a previous study by Peter et al., wherein the corrosion current density from crystalline BMG was slightly greater than the non-crystalline structure. Surface oxide layers can exhibit different potentials than the base metal, and thus affect corrosion behaviour. For example, the standard reversible electrode potential for titanium is negative, but in practice, the electrode potential of titanium in the galvanic series is positive because of the passive oxide layer on the surface.

## 4. Conclusions

For the first time in a basic medium, the *Aerva-lanata* flower was successfully employed as a green corrosion inhibitor for carbon-fibre-and-aluminium-metal-based fibre–metal laminates. In fact, the flower of aerva lanata contains aromatic rings, heteroatoms, and oxygen, which makes it a suitable candidate for acting as an inhibitor in the basic medium. The inhibition efficiency reached a maximum of about 92 percent with electrochemical techniques in the presence of 600 ppm inhibitor. SEM analysis was used to analyse the surface morphology of the carbon-fibre-and-aluminium-metal-based fibre–metal-laminate microstructure in the absence and presence of the aerva-lanata-extract inhibitor. The Langmuir adsorption isotherm was detected as the inhibition mechanism. Due to the complex protective film formation between the inhibitor and the metal surface ions, there was a decrease in charge and ion transfer on the metal surface, which was recognised as the reason for the existence of the inhibition property.

## Figures and Tables

**Table 1:** Inhibition Efficiency Developed from Different Plant Extracts.

Plant Name	Inhibition Efficiency	Material Used	Medium
Saraka Ashoka	95.48	carbon steel	0.5 M H_2_SO_4_
Chitosan	92%	300 mg/L on mild steel	1M sulfamic acid
Glycyrrhiza glabra leaves	88%	800 ppm on mild steel	1 M HCl
Sunflower-seed hull	98%	300 ppm on aluminium	1 M HCl
Pyridazinium	84%	100 mg/L on mild steel	1 M HCl
Pyrazolo-pyridines	97%	100 mg/L on carbon steel	1 M HCl
Salvia officinalis	96%	2500 mg/L on stainless steel	HCl
Osmanthus fragran	94%	340 mg/L on carbon steel	HCl
Musa paradisica	90%	300 mg/L on carbon steel	HCl
Mangrove tannins	89%	6000 mg/L on metal	Acidic medium
Jasminumnudiflorum	92%	1000 mg/L on aluminium	HCl
Lawsonia inermis	92%	1200 mg/L on moderate steel	1 m HCl
Dendrocalamus brandisii	90%	1000 mg/L on aluminium	HCl, H_3_PO_4_
Aqueous coffee grounds	83%	400 mg/L on carbon steel	1 M HCl
Phyllanthus amarus	81%	4000 mg/L on mild steel	Acidic media
Black radish	92%	1000 mg/L on carbon steel	-
Ginkgo	80%	100 mg/L on carbon steel	HCl and H_2_SO_4_

**Table 2:** Weight-Loss-Method Calculation of FML in 3.5% NaCl.

Elemental Composition	% of Element
Manganese (Mn)	0.0–0.15
Iron (Fe)	0.0–0.70
Magnesium (Mg)	0.80–1.20
Silicon (Si)	0.40–0.80
Copper (Cu)	0.15–0.40
Zinc (Zn)	0.0–0.25
Titanium (Ti)	0.0–0.15
Chromium (Cr)	0.04–0.35
Other (Each)	0.0–0.05
Others (Total)	0.0–0.15
Aluminium (Al)	Balance

**Table 3:** Weight-Loss-Method Calculation of FML in 3.5% NaCl.

Days	Weight of the Bare FML (g)	Corrosion Rate (mmpy)	Weight of the Coated FML (g)	Corrosion Rate (mmpy)	Efficiency (%)
Initial	1.5394	-	1.8249	-	-
1	1.5250	3.5358	1.8230	0.4665	86.8
2	1.5079	4.1982	1.8210	0.4912	88.29
3	1.4924	3.8065	1.8189	0.5056	86.71
4	1.4768	3.8312	1.8168	0.5147	86.56
5	1.4608	3.9287	1.8147	0.5184	86.8
Average					87.032

**Table 4:** Weight of the Specimen before and after Coating.

Concentration (ppm)	Before Coating (g)	After Coating (g)
Bare	1.5394	-
600	1.5346	1.8890

**Table 5:** Polarization parameters for FML in NaCl.

Specimen	E_corr_ (V)	I_corr_ (A cm^−2^)	Efficiency (η %)
Brae FML	−0.79	0.001959	-
Coated FML	−0.77	0.0002335	88

**Table 6:** EIS parameters for FML in NaCl.

Specimen	Rct (Ω cm^2^)	CPE (μF cm^−2^)	n	Efficiency (η %)
Brae FML (0)	300.23	1.09 × 10^−3^	0.0715	-
Coated FML (150)	301.15	1.72 × 10^−3^	0.0785	85.9

**Table 7:** Vickers hardness values for the FML at various conditions.

Specimen	Applied Force (P) in kg	Vickers Hardness Number (VHN) VHN = (1824 × P)/d^2^			Average Vickers Hardness Number	Standard Deviation
		Trial 1	Trial 2	Trial 3		
Coated FML after immersion	30	293	307	297	299	5.89
Bare FML after immersion	30	211	215	201	209	5.89
Untreated FML	30	530	518	518	522	5.66

## Data Availability

Data is contained within the article.
